# Sensory processing and eating behaviours in autism: A systematic review

**DOI:** 10.1002/erv.2920

**Published:** 2022-06-23

**Authors:** Emy Nimbley, Lisa Golds, Helen Sharpe, Karri Gillespie‐Smith, Fiona Duffy

**Affiliations:** ^1^ School of Health in Social Sciences University of Edinburgh Edinburgh UK; ^2^ NHS Lothian Child and Adolescent Mental Health Services Royal Edinburgh Hospital Edinburgh UK

**Keywords:** autism, eating behaviours, sensory processing, sensory sensitivities

## Abstract

**Objectives:**

The aim of this study was to assess the relationship between sensory processing and a broad range of eating behaviours across the lifespan.

**Methods:**

Five electronic databases of published and unpublished quantitative studies were systematically searched, evaluated for risk of bias and synthesised according to identified eating outcomes.

**Results:**

Across 25 studies, there was consistent evidence of a relationship between sensory processing and a range of eating behaviours. There was early evidence for the particular role of taste/smell sensitivities, as well as hypersensitivities, although future research is needed looking at different sensory patterns and modalities. There was also tentative evidence to suggest this relationship extends across development.

**Discussion:**

Study findings are discussed in relation to implications for sensory‐based eating and feeding interventions and the development of eating disorders. Methodological and conceptual limitations are discussed and suggestions for future research are made to address these limitations. A broader investigation of multi‐sensory issues and clearly defined eating behaviours, including disordered eating in clinically diagnosed samples, will allow for a more comprehensive and robust understanding of the relationship between sensory processing and eating behaviours in autism.

## Key points

- Sensory processing, notably taste/smell and hypersensitivities, was associated with a broad range of eating behaviours in autism, although no study looked at disordered eating outcomes.
- There are clear implications for the development of sensory‐based eating interventions in clinical and subclinical populations, highlighting the need for considering and adjusting for unique sensory needs in treatment approaches.
- A broader investigation of different sensory profiles and disordered eating outcomes across development will allow us to untangle the role of sensory processing in autism and eating behaviours.

## 1 INTRODUCTION

Autism spectrum disorder (ASD), here on referred to as autism, is clinically characterised by differences in social interaction and communication, the presence of restrictive and repetitive behaviours and differences in sensory processing (APA, 2013). Sensory differences reflect a heterogenous presentation of global and multi‐modal differences and have been reported in up to 90% of individuals (Baranek et al., 2006; De Both & Reynolds, 2015). The DSM‐V (APA, 2013) adopts one of the most common theoretical approaches to sensory processing that highlights patterns of hyper‐ and hypo‐sensitivities (Boyd et al., 2009; Marco et al., 2011) as well as sensory seeking behaviours (Ben‐Sasoon et al., 2009). These subtypes can refer to sensitivities at an individual sensory modality but are also hypothesised to apply across multiple sensory modalities (Tomchek et al., 2007; Crasta et al., 2020). This latter conceptualisation of sensory processing is in line with emerging evidence for differences in integrating sensory information across modalities in autism (Iarocci & Donald, 2006; Stevenson et al., 2015). It is now generally accepted that sensory differences are integral in our understanding of autism, not just as core symptoms but also in their influence on different behavioural and clinical features (Glod et al., 2015; Robertson & Baron‐Cohen, 2017).

While atypical eating behaviours are reported to be common in neurotypical childhood (Dovey et al., 2008; Dubois et al., 2008; Micali et al., 2011), such behaviours appear to be a more frequent and persistent issue in autism (Bandini et al., 2017; Baraskewich et al., 2021; Sharp et al., 2013; Twachtman‐Reilly et al., 2008). Clinicians and caregivers frequently report a broad range of such behaviours in autistic individuals, such as food selectivity, disruptive mealtime behaviours and food neophobia, here defined as a fear of trying new foods (Edmond et al., 2010; Margari et al., 2020; Wallace et al., 2018), aversions have also been cited based on sensory characteristics or features of foods, such as texture, colour or specific brands (Ahearn et al., 2001; Schrek et al., 2004; Schrek & Williams, 2006). Subsequent empirical studies have implicated these food‐specific sensory characteristics in autistic individuals who demonstrate atypical eating behaviours (Hubbard et al., 2014; Kuschner et al., 2015; Postorino et al., 2015), leading to the proposal that differences in sensory processing may account for these aversions, and may in turn be an underlying mechanism of atypical eating behaviours in autism (Mari‐Bauset et al., 2014; Cermak et al., 2010).

The clinical implications of this relationship are significant. Not only are there associated health risks, such as weight concerns and gastrointesintal (GI) issues (Brown et al., 2016), but there is also increasing evidence suggesting a heightened risk of developing eating and feeding disorders in autism (Bourne et al., 2021; Gesi et al., 2021; Tchanturia et al., 2019; Westwood & Tchanturia, 2017). The trajectory and underlying mechanisms of this relationship remain largely unknown. Recent longitudinal studies report that greater autistic traits in childhood predict disordered eating in adolescence (Solmi et al., 2021), with fussy eating habits in childhood identified as a possible mediator (Leno et al., 2022). This would suggest that autism could be a risk factor in the development of disordered eating, and that atypical eating behaviours in childhood particularly act as a precursor to the development of eating or feeding issues. Alternatively, there could be common underlying vulnerability mechanisms, such as sensory processing (Brede et al., 2020; Kinnaird et al., 2018).

Two previous reviews have addressed the relationship between sensory processing and eating behaviours in autism. In a systematic review looking at a range of psychological correlates of sensory processing, Glod et al. (2015) identified one study exploring eating behaviours that did not find evidence to support a relationship. More recently, Page et al. (2021) reviewed correlates of childhood eating and feeding difficulties in autism and found that atypical eating behaviours, particularly food selectivity, are consistently correlated with differences in sensory processing. The current review seeks to extend these reviews by adopting a lifespan approach to the relationship between sensory processing and eating behaviours. The current review also seeks to account for a wide range of clearly defined and conceptualised eating behaviours following calls to address significant heterogeneity in definitions of eating behaviours and criticism of previous measures in their neglect of important autism‐specific behaviours associated with the mealtime environment (Bandini et al., 2017; DeMand et al., 2015; Page et al., 2021). Therefore, this review will aim to identify, evaluate and synthesise up‐to‐date literature to provide an evidence‐based answer to the following research question: is there a relationship between sensory processing and eating behaviours in autism across the lifespan?

## 2 METHODS

This review was conducted in line with the Preferred Reporting Items for Systematic Reviews and Meta‐Analyses (PRISMA) statement (PRISMA; Page et al., 2020).

### 2.1 Eligibility criteria

Studies were eligible for inclusion if they looked at the relationship between sensory processing and eating behaviours in individuals with a diagnosis of autism, in both clinical and community populations. Full text was required to be available in English. Both published and unpublished studies were included, with no exclusion based on publication date. Studies were also required to report a measure of effect of the relationship between sensory processing (considered here as the exposure) and eating behaviours (considered here as the outcome).

For the purposes of this review, *sensory processing* was defined as sensory sensitivities, such as modality‐specific as well as interoceptive, proprioceptive and vestibular hyper‐ and hypo‐sensitivities, as well as sensory seeking and multi‐sensory integration (Ben‐Sassoon et al., 2009; Mari‐Bauset et al., 2014). Studies were included if they used a measure of sensory processing in line with this definition. These measures could be observational, behavioural or physiological, and could involve self‐, parent‐ and/or clinician‐report. Studies that looked at sensory characteristics of food (e.g., consistencies, brand, foods touching other foods, etc.) or cognitive or affective processing of food or food‐related stimuli (e.g., emotional/hedonic ratings or responses to food) *only*, without linking these processes to general sensory modalities or patterns were not included. *Eating behaviours* was here defined as any eating or feeding behaviours, including mealtime behaviours, food selectivity, food neophobia or disordered eating (Margari et al., 2020; Martins et al., 2008). Studies were included if they used a measure of eating behaviours in line with this definition. These measures could be observational or behavioural, and could involve self‐, parent‐ and/or clinician‐report. Studies were excluded if they reported on dietary‐related conditions *only*. No exclusions were made with regard to age, gender or comorbidity.

### 2.2 Information sources and search strategy

Following a scoping search, five databases were searched in March 2021: psychINFO (OVID), Scopus, PubMed and Web of Science were used to search for published studies, while ProQuest Dissertation and Theses for unpublished studies. Search terms were identified from early scoping searches and in collaboration with information specialists at the University of Edinburgh and included autism AND sensory processing OR hypersensitivities OR hyposensitivities AND eating OR feeding OR food OR mealtime.

### 2.3 Selection and data collection process

Two reviewers (Emy Nimbley and Lisa Golds) independently screened title and abstracts, with the LG screening two‐thirds (66%). Full texts of potentially eligible studies were retrieved where possible and screened against inclusion criteria. Any full texts that did not meet inclusion criteria were excluded. The following data were extracted from each paper by Emy Nimbley: study characteristics (e.g., population, setting, power and/or sample calculations, missing data computations, adjustment for confounders), participant characteristics (e.g., means and standard deviations of age, percentage of gender, percentage ethnicity) and measures of interest (e.g., measures of relationship between sensory processing and eating behaviours). Where more than one paper was published from the same study, data was extracted in chronological order, starting with primary publication and updating relevant data where appropriate.

### 2.4 Study risk of bias assessment

Quality assessment based on the NIH Quality Assessment Tool for Observational, Cross‐Sectional and Cohort Studies was conducted by Emy Nimbley and Lisa Golds on included studies. Inter‐rater agreement was 87%, and agreement was reached on the remainder through mutual agreement.

## 3 RESULTS

**FIGURE 1:**
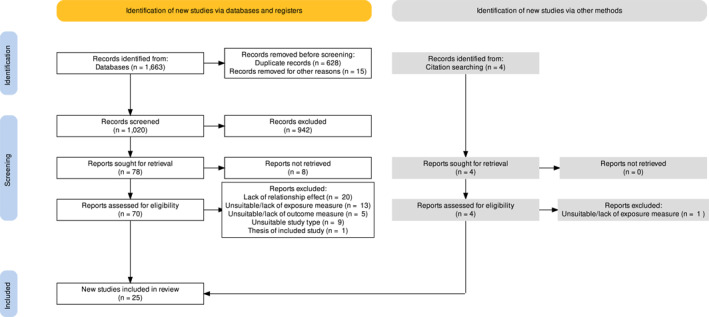
Preferred reporting items for systematic reviews and meta‐analyses flow diagram depicting the screening and selection process of the current review

Online database searches generated 1663 studies that were screened for eligibility. Following screening of titles and abstracts, 71 studies were screened at full text. The selection and screening process resulted in 26 papers reporting 25 independent studies to be included in the current study. Four studies were identified by the process of backward citation chaining. See the PRISMA flow diagram depicting the screening and selection process for included studies (Figure 1).

### 3.1 Study characteristics

Of the 25 studies included in this review, 24 had a cross‐sectional design and one study had a longitudinal design (see Table 1). Two of the studies conducted a secondary data analysis, and the remaining 24 studies utilised primary data. All studies were published between 2008 and 2021, with most studies conducted in the US (*n* = 11). Across studies, there was a total of 4338 participants included, with sample sizes ranging from 25 to 1112 (median *n* = 98). Participants ages ranged from 1 to 28 years, with the majority looking at the relationship between sensory processing and eating behaviours during childhood (*n* = 18). Stage of childhood varied considerably across studies, and there was a notable lack of definition or justification of chosen age range. Twenty‐four studies look at mixed‐gender samples, and one study looked at a female only sample.

**TABLE 1:** Study characteristics

Author	Country	Design	Control group	*N*	Developmental stage (range)	*M* (SD) in years	% Female
Bitsika and Sharpley (2018)	Australia	Cross‐sectional	None	37	Childhood (6–11 years)	8.5 (1.5)	100%
Chistol et al. (2018)	US	Cross‐sectional	Age‐matched NT	53 AUT	Childhood (3–11 years)	6.6 (2.1) AUT	17% AUT
				56 NT		6.7 (2.4) NT	22% NT
Crasta et al. (2014)	India	Cross‐sectional	ID	41 AUT	Childhood (3–10 years)	6.42 (1.92) AUT	26%^a^
				56 ID		7.15 (1.76) NT	
Johnson et al. (2014)	US	Cross‐sectional	None	256	Childhood (2–11 years)	5.4 (2.4)	16%
Koshy (2015)	UK	Cross‐sectional	None	144 ASD	Childhood and adolescence (2–18 years)	5.2 ASD	18.2% ASD
				391 autism		4.2 autism,	18.8% autism
				123 asperger		7.4 asperger	12.2% asperger
Kral et al. (2015)	US	Cross‐sectional	NT	25 AUT	Childhood (4–6 years)	5.0 (0.9) AUT	28% AUT
				30 TD		5.2 (0.7) NT	53% NT
Lane et al. (2014)	Australia	Cross‐sectional	None	36	Childhood (3–10 years)	6.7	20%
Leader et al. (2020)	Ireland	Cross‐sectional	None	136	Childhood and adolescence (ns)	8.46 (4.13)	28%
Leader et al. (2021)	Ireland	Cross‐sectional	None	120	Childhood and adolescence (ns)	8.0 (3.79)	22.5%
Martins et al. (2008)	Australia	Cross‐sectional	NT, SIB	41 AUT	Childhood (2–12 years)	‐	17% AUT
				14 SIB			50% SIB
				41 NT			44% NT
Nadon et al. (2011)	Canada	Cross‐sectional	None	95	Childhood (3–10 years)	7.3 (2.4)	8.4%
Padmanabhan and Schroff (2020)	India	Cross‐sectional	None	146	Childhood (3–11 years)	7.09 (2.55)	19%
Panerai et al. (2020)	Italy	Cross‐sectional	None	111	Childhood (2–12 years)	*Median, split into:*	*Split into:*
						5.0 AUT	18.9% AUT‐W
						5.3 NT	24.3% aut‐wo
Pomoni (2016)	UK	Cross‐sectional	NT	103 AUT	Childhood (2–14 years)	7.32 (2.58) AUT only	22.3% AUT
				151 NT			51.0% NT
Riccio et al. (2018)	Italy	Cross‐sectional	Age‐matched NT	43 AUT	Childhood (2–11 years)	6.28 (2.3) AUT 7.2 NT	30% AUT
				41 NT			
Schnizler (2014)	US	Cross‐sectional	Age‐ and IQ‐matched NT	28 AUT	Childhood and adolescence (2–18 years)	12.75 (1.90) AUT	21.4% AUT
				31 NT		13.07 (2.92) NT	45.2% NT
Shamaya et al. (2017)	Israel	Cross‐sectional	Age‐ and gender‐matched NT, SIB	50 AUT	Childhood (3–6 years)	*In months:*	19.6% AUT
				12 SIB		5.41 (11.1) AUT	28.6% SIB
				29 NT		77 (32) SIB	24.1% NT
						51.6 (11.6) NT	
Smith et al. (2020)	UK	Cross‐sectional	NT, TS, ADHD	27 AUT	Childhood and adolescence (6–17 years)	‐	40.7% AUT
				27 NT			18.5% NT
				27 TD			18.5% TS
				17 ADHD			41.2% ADHD
Suarez et al. (2012, 2014)	US	1. Cross‐sectional 2. Longitudinal	None	1. 141	Childhood (3–9 years)	1. *Split into:*	1. 5%
				2. 54		6.23 (1.85) severe	2. 12%
						6.24 (1.39) moderate	
						6.88 (1.51) typical	
						2. ‐	
Tanner et al. (2015)	US	Cross‐sectional	None	35	Childhood (4–10 years)	*Split into:*	*Split into:*
						6.5 (1.9) selective	11.8% selective
						6.9 (2.0) nonselective	5.6% nonselective
Trinh (2014)	US	Cross‐sectional	NT	9 AUT	Childhood (5–12 years)	9.11 (0.72) AUT	‐
				16 NT		7.19 (0.53) NT	
Wang et al. (2019)	China	Cross‐sectional	Age‐matched NT	81 AUT	Childhood (3–7 years)	5.18 (0.92) AUT	17.3% AUT
				153 NT		5.34 (1.14) NT	16.8% NT
Zickgraf and Mayes (2018)	US	Cross‐sectional	None	1112	Childhood and adolescence (1–17 years)	6.4 (3.3)	10.6%
Zickgraf et al. (2020)	US	Cross‐sectional^b^	None	185	Childhood, adolescence and young adulthood (4–17 years)	8.65 (3.03)	28%
Zobel‐Lachuisa et al. (2015)	US	Cross‐sectional	NT	34 AUT	Childhood (5–12 years)	8.61 (2.32) AUT	3% AUT
				34 NT		8.76 (2.23) NT	20.6% NT

Abbreviations: ADHD, attention deficit hyperactivity disorder; ASD, autism spectrum disorder; AUT, autism; AUT‐W, autism with feeding problems; AUT‐WO, autism without feeding problems; DD, developmental delay; ID, intellectual disability; NT, neurotypical; OCD, obsessive compulsive disorder; REP, representative sample; SIB, neurotypical siblings of an autistic child; TS, Tourette's syndrome; UG, undergraduate sample.

^a^ Total sample.

^b^ Secondary data analysis.

### 3.2 Risk of bias

Out of the 25 studies included, six were deemed good quality, four were deemed poor quality and the remaining 15 were deemed fair quality (see Table 2). Eight of the included studies reported that participation rates of eligible persons were at least 50% and nine studies failed to report full inclusion and exclusion criteria, suggesting risk of selection and response bias. Due to only one study being a longitudinal design, there was a consistent lack of sufficient timeframe to reasonably expect to see an association between variables. Furthermore, three studies did not use a validated measures of sensory processing and six studies did not use a validated measure of eating behaviours, suggestive of a measurement bias, particularly regarding eating outcome measures. Additionally, all unpublished studies reported significant results, suggesting there was no evidence of publication bias.

**TABLE 2:** Quality assessment and risk of bias

	Research question or aims/objectives clearly stated?	Study population clearly and specifically defined?	Participation rate of eligible participants at least 50%?	All subjects recruited from same/similar populations?	Inclusion and exclusion criteria prespecified and uniformly applied?	Sample size, power or variance and effect estimates?	Exposure measured prior to outcome?	Sufficient timeframe to reasonably expect association?	Exposure clearly defined, valid, reliable and implemented consistently?	Exposure assessed more than once?	Outcome clearly defined, valid, reliable and implemented consistently?	Outcome assessors blind to exposure status?	Loss to follow‐up 20% or less?	Key confounding variables measured and controlled for?	Overall quality of study
Bitsika and Sharpley (2018)	‐	+	NR	+	NR	‐	‐	‐	+	‐	‐	‐	NA	+	Poor
Chistol et al. (2018)	+	+	NR	+	+	+	‐	‐	+	‐	+	‐	NA	+	Good
Crasta et al. (2014)	‐	+	+	+	CD	+	‐	‐	+	‐	+	+	NA	+	Fair
Johnson et al. (2014)	+	+	+	+	CD	‐	‐	‐	+	‐	+	‐	NA	+	Fair
Koshy (2015)	+	+	‐	+	+	+	‐	‐	+	‐	+	‐	NA	+	Fair
Kral et al. (2015)	+	+	NR	+	+	‐	‐	‐	+	‐	+	‐	NA	+	Fair
Lane et al. (2014)	+	+	NR	+	NR	‐	‐	‐	+	‐	+	‐	NA	+	Fair
Leader et al. (2020)	+	+	NR	+	NR	‐	‐	‐	+	‐	+	‐	NA	+	Fair
Leader et al. (2021)	+	+	NR	+	NR	‐	‐	‐	+	‐	+	‐	NA	+	Fair
Martins et al. (2008)	+	+	+	+	+	‐	‐	‐	‐	‐	CD	‐	NA	‐	Poor
Nadon et al. (2011)	+	+	+	+	+	‐	‐	‐	+	‐	+	‐	NA	+	Fair
Padmanabhan and Schroff (2020)	+	+	NR	+	+	+	‐	‐	+	‐	+	‐	NA	‐	Good
Panerai et al. (2020)	+	+	NR	+	+	+	‐	‐	+	‐	+	‐	NA	+	Good
Pomoni (2016)	+	+	NR	+	+	‐	‐	‐	+	‐	+	‐	NA	+	Fair
Riccio et al. (2018)	+	+	NR	+	+	‐	‐	‐	+	‐	+	‐	NA	‐	Fair
Schnizler (2014)	+	+	NR	NR	NR	‐	‐	‐	+	‐	+	‐	NA	+	Poor
Shamaya et al. (2017)	+	+	+	+	+	+	‐	‐	+	‐	+	‐	NA	+	Good
Smith et al. (2020)	+	+	+	+	+	‐	‐	‐	+	‐	+	‐	NA	+	Good
Suarez et al. (2012, 2014)	+	+	‐	+	+	‐	‐	+	‐	‐	+	‐	‐	+	Fair
Tanner et al. (2015)	+	+	+	+	+	+	‐	‐	+	‐	+	‐	NA	+	Good
Trinh (2014)	+	+	NR	+	+	+	‐	‐	+	‐	+	‐	NA	+	Fair
Wang et al. (2019)	+	+	NR	+	+	+	‐	‐	+	‐	+	‐	NA	+	Fair
Zickgraf & Mayes (2018)	+	+	+	‐	+	+	‐	‐	‐	‐	‐	‐	NA	+	Poor
Zickgraf et al. (2020)	+	+	NR	NR	NR	‐	‐	‐	‐	‐	‐	‐	NA	+	Poor
Zobel‐Lachuisa et al. (2015)	+	+	NR	+	+	+	‐	‐	+	‐	+	‐	NA	+	Fair

*Note*: +, Yes; ‐, No; CD, cannot determine; NR, not reported; NA, not applicable.

### 3.3 Study results

Key results of included studies are presented in Table 3. The following section will synthesise the results summarised by eating outcome. There was notable heterogeneity within studies as to standardized definitions of eating outcomes. In some cases, food selectivity is used to describe a singular behaviour indicative of picky eating, while in others it was used interchangeably with terms such as food refusal or limited repertoire. Attempts towards a standardized definition acknowledge these latter terms of selective behaviours as two separate and empirically measurable domains (Bandini et al., 2010, 2017) leading to recent calls for defining and measuring each domain individually (Page et al., 2021). Thus, for the purposes of this review, they will be treated as distinct categories in instances whereby these separate behaviours can be isolated; food refusal behaviours will fall under the umbrella category of *Mealtime Behaviours*, while *Food Repertoire* will form a category of its own. In instances whereby these two domains cannot be isolated, food selectivity will be used to describe the singular behaviour of fussy eating and will similarly be included under the umbrella category of *Mealtime Behaviours.* Thus, the following section will qualitatively synthesise results by the following eating outcomes:

- *Mealtime Behaviours*, which here refers to outcomes associated with behaviours typically displayed around mealtimes, such as general mealtime and eating behaviours, food refusal, food selectivity or fussiness, ritualistic eating behaviours and over‐ or under‐eating
- *Food Repertoire*, which here refers to food or dietary intake; and
- *Food Neophobia*, which here refers to fear of trying new foods. No study that met inclusion criteria explored disordered eating as an outcome.

**TABLE 3:** Study results

Author	*N* =	Sensory measure	Sensory measure collection method	Eating measure	Eating measure collection method	Controlled for	Other measures	Relationship findings
Bitsika and Sharpley (2018)	37	SP	Parent‐report	SWEAA	Parent‐report	Medication, comorbidities	WASI‐II, ADOS‐2, SRS	Two SRS RRB subscales only variable that sig predicted eating disturbances
Chistol et al. (2018)	56 AUT	SP	Parent‐report	FFQ, 3‐day food record	Parent‐report	Age, sex, race/ethnicity, only child	VABS, DAS	Autistic children with oral sensitivities exhibited more food refusal and less vegetables; oral over‐sensitivities associated with food refusal, limited repertoire and limited fruit/vegetable
	56 NT							
Crasta et al. (2014)	41 AUT	SP	Parent‐report	BAMBI	Parent‐report	Gender, IQ, comorbidity	CARS, BKSI/GDS, VSMS	Food refusal, disruptive mealtime behaviour and limited repertoire sig associated with sensory processing across domains; food selectivity associated with oral only
	56 ID							
Johnson et al. (2014)	256	SSP	Parent‐report	BAMBI, HEI	Parent‐report	Medication	ADOS, MSEL/SB:5th, RBSR, CBCL	Predictive relationship of sensory processing on eating behaviours
Koshy (2015)	639	SSP	Parent‐report	ASD‐CC	Parent‐report	Age	ASD‐PBC	Sig association between both under‐ and over‐eating and sensory processing across
Kral et al. (2015)	25 AUT	SP	Parent‐report	CFNS, CEBQ	Parent‐report	Age, sex, race/ethnicity height, weight, BMI	SCQ, CFQ, PFSQ	AUT: Atypical oral sensory sensitivity showed higher food neophobia and fussiness
	30 NT							
Lane et al. (2014)	30	SP	Parent‐report	BAMBI, 3‐day food record	Parent‐report			Sig, association between taste/smell sensitivities and both food refusal and limited repertoire
Leader et al. (2020)	136	SSP	Parent‐report	STEP‐CHILD	Parent‐report	Age, gender, GI symptoms, challenging behaviour, comorbid psychopathology	GI symptom inventory, BPI‐S, ASD‐CC	Sig relationship between sensory processing and rapid eating, food refusal, food selectivitySig predictive relationship of sensory processing for food selectivity
Leader et al. (2021)	120	SSP	Parent‐report	STEP‐CHILD	Parent‐report	Age, gender, GI symptoms, challenging behaviour, comorbid psychopathology, adaptive functioning, quality of life	GI symptom inventory, BPI‐S, ASD‐CC, pedsql, VABS‐II, CAM treatments	Sig relationship between sensory processing and: chewing problems, particularly sensory seeking; rapid eating, particularly auditory; food selectivity.
								Sig predictive relationship of sensory processing for food refusal
Martins et al. (2008)	41 AUT	CARS	Parent‐report	Eating behaviours questionnaire, BPFAS, FNS	Parent‐report		BPFAS(PEB), VABS	AUT: Sig relationship between sensory processing and ritualistic feeding behaviour
	14 SIB							
	31 NT							
Nadon et al. (2011)	95	SSP	Parent‐report	Eating profile	Parent‐report			Sig, predictive relationship between sensory processing and eating problems, particularly taste/smell and visual/auditory; tactile sig correlated but not predictive
Padmanabhan and Schroff (2020)	146	SSP	Parent‐report	BAMBI, 24 h dietary recall	Parent‐report		BMI	Sig relationship between sensory processing and eating behaviours; taste/smell sig associated with mealtime behaviours and food refusal
Panerai et al. (2020)	111	SSP, SEQ	Parent‐report	CEBQ, BAMBI	Parent‐report	Age, gender, autism severity	ADI‐R/CARS	Group differences = AUT with feeding problems more likely to have multi‐sensory issues (taste/smell, tactile, sensory seeking, auditory, low energy).
								Sig correlations between sensory processing and general eating behaviours
Pomoni (2016)	103 AUT	SSP	Parent‐report	FBS	Parent‐report	Behavioural/emotional problems, social and communication skills	SCQ, SDQ	Sig relationship between sensory processing and eating behaviours in both groups, across modalities; stronger in AUT group
	151 NT							
Riccio et al. (2018)	43 AUT	TASR38 genotype	Biological	FPI	Parent‐report		ADOS‐2, GMDS‐ER, Leiter‐R, ADI‐R, CARS, VABS	AUT: Sig relationship between food selectivity and bitter taste sensitivity genotype in AUT
	41 NT							
Schnizler (2014)	28 AUT	EHS	Parent‐report	EHS	Parent‐report	Age, IQ, gender, behavioural flexibility	ADI, ADOS, WISC‐III, flexibility scale (routines/Rituals subscale)	Oral sensitivities emerged as sole predictor; olfactory sensitivities and behavioural flexibility ns predictors
	31 NT							
Shamaya et al. (2017)	50 AUT	SP	Parent‐report	BAMBI, 3‐day food record	Parent‐report		BMI	AUT: Sig association between multi‐modal sensory processing (factors, quadrants, sections) and mealtime behaviours, food refusal and limited variety; oral sensitivities implicated
	12 SIB							
	29 NT							
Smith et al. (2020)	27 AUT	SSP	Parent‐report	FPQ, CEBQ (food fussiness subscale)	Parent‐report	Age, gender, diagnosis	BMI	Taste/smell sensitivities only sig predictor of eating behaviours across all neurodevelopmental disorders; fully mediated food fussiness differences compared to TD
	27 TS							
	17 ADHD							
	27 NT							
Suarez et al. (2012, 2014)	1. 1412. 54	SOR (composite score from SSP and red flags of sensory over‐responsivity	Parent‐report	Singe item from online survey	Parent‐report	1. Age, physiological factors	1. Online survey	1. Sig relationship between sensory over‐responsivity only and food selectivity, particularly tactile sensitivities
						2. Age, RRBs	2. Online survey, RBS‐R	2. Sig relationship consistent over time, only predictive variable (not RRBs)
Tanner et al. (2015)	35	SSP	Parent‐report	FFQ, BAMBI	Parent‐report	Age, gender, parent sex, race/ethnicity, food security status, BMI, social communication	SCQ, CBCL, RBS‐r	Group differences = ns on sensory processing (taste/smell) in selective versus non‐selective.
								Sig relationship between taste/smell sensitivities and food refusal and food repertoire (limited variety, total foods eaten)
Trinh (2014)	9 AUT	Composite scores: SSP and four‐item texture problems	Parent‐report	BAMBI, food preferences inventory	Parent‐report	Age, diagnosis, coping skills, expressive language skills, parent feeding practices, parent feeding practices	ERC, VABS, parent mealtime action scale	Sensory processing found to sig predict mealtime behaviours in total sample (both AUT and NT)
	16 NT							
Wang et al. (2019)	81 AUT	SSP	Parent‐report	MBQ	Parent‐report	Age, gender, autism severity, receptive vocabulary, sleep, emotional and behavioural problems	SCQ, CARS, CHSQ, SDQ, PPVT‐C	Sig association between sensory processing and mealtime behaviours in both groups; taste sensitivity particularly implicated in AUT group
	153 NT							
Zickgraf and Mayes (2018)	1112	CASD	Clinician‐report	CASD	Clinician‐report	Age, gender, IQ, medication, comorbid psychopathology	PBS	Tactile processing differences more common in those with atypical eating behaviours, but was ns in predicting atypical eating behaviours
Zickgraf et al. (2020)	185 AUT	EHS (AUT only)	Parent‐report	CEBQ (food fussiness scale) (AUT only)	Parent‐report	Age, gender, rigidity	Flexibility scale‐revised	Oral sensitivities sig predictor of selective eating (smell ns)
	179 OCD							
	263 REP							
	813 UG							
Zobel‐Lachuisa et al. (2015)	34 AUT	SSP, SEC; TIE	Parent‐report;	BAMBI	Parent‐report	Age		Sig relationship between all sensory measures and mealtime behaviours; sig relationship between all sensory subscales and mealtime behaviours
	34 NT		Child‐report					

Abbreviations: ABAS‐II, adaptive behaviour assessment system‐II; ADI‐R, autism diagnosis interview‐revised; ADOS, autism diagnostic observation schedule; ADOS‐2, Autism diagnostic observation schedule second edition; ASD‐CC, autism spectrum disorder‐comorbidity for children; ASD‐PBC, autism spectrum disorder‐problem behaviour for children; BAMBI, brief autism mealtime behaviour inventory; BKIS, binet kamat scale of intelligence; BPFAS(PEB), parent section of behavioural paediatric feeding assessment scale; BPFAS, behavioural paediatric feeding assessment scale; BPI‐S, behaviour problems inventory‐short form; CAM, complementary/alternative medicine; CARS, childhood autism rating scale; CASD, checklist for autism spectrum disorders; CBCL, child behaviour checklist; CEBQ, child eating behaviour questionnaire; CFNS, child food neophobia scale; CFQ, child feeding questionnaire; CHSQ, Chinese sleep habits questionnaire; DAS, differential abilities scales; EHS, eating habits survey; ERC, emotion regulation checklist; FBS, feeding behaviour checklist; FFQ, food frequency questionnaire; FPI, food preferences inventory; FPQ, food preferences questionnaire; GMDS‐R, griffiths mental development scales; HEI, healthy eating index; Leiter‐R, lieter international performance test‐revised; MBQ, mealtime behaviour questionnaire; MSEL, mullen scales of eating learning; PBS, paediatric behaviour scale; PedsQL, paediatric quality of life inventory‐fourth version; PFSQ, parental feeding style questionnaire; PPVT‐C, peabody picture vocabulary test‐Chinese edition; RBSR, repetitive behaviour checklist; RBS‐R, repetitive behaviours scale‐revised; SB:fifth, stanford‐binet fifth edition; SCQ, social communication questionnaire; SDQ, strengths and difficulties questionnaire; SEQ, sensory experiences questionnaire; SOR, sensory over‐responsivity scale; SP, sensory profile; SRS, social responsiveness scale; SSP, short sensory profile; STEP‐CHILD, screening tool of feeding problems, for children; SWEAA, Swedish eating assessment for autism spectrum disorders; TIE, touch inventory for elementary‐school‐aged‐children; VABS, vineland adaptive behaviour scales; VABS‐II, vineland adaptive behaviour scale‐second edition; VSMS, vineland social maturity scale; WASI‐II, Weschler abbreviated scale of intelligence second edition.

With regards to sensory processing, it is also important at this stage to clarify possible heterogeneity. 'Oral sensory processing’ refers to the processing of food or other objects put in the mouth, and oral sensory sensitivities refer to atypical sensory processing associated with this process (Chaware et al., 2021). Oral sensory sensitivities can encompass atypical processing across multiple sensory domains, including taste, smell and tactile processing, and thus have been synthesised as a distinct sensory domain than taste/smell sensitivities alone.

## 4 MEALTIME BEHAVIOURS

### 4.1 General mealtime and eating behaviours

Eleven studies looked at general mealtime behaviours. Three studies looked at the relationship between general sensory processing and mealtime behaviours. Johnson et al. (2014) and Zobel‐Lachuisa et al. (2015) reported significant correlations between sensory processing and mealtime behaviours in autistic children, with the latter study also providing evidence to suggest that this association is stronger in autism compared to neurotypical peers (*r* = 0.378–0.747 vs. *r* = 0.153–0.622). Sensory processing difficulties were found to significantly predict mealtime behaviours in Johnston et al. (2014) and Trinh (2014) studies, however, there was no comparison or control group included. Furthermore, Trinh (2014) used a mixed autism‐neurotypical sample, and therefore no autism‐specific conclusions can be drawn. Collectively, these studies offer preliminary support of a relationship between general sensory processing issues and mealtime behaviours in autistic children. These studies offer preliminary support of a relationship between general sensory processing issues and mealtime behaviours in autistic children that warrants future investigation to determine whether how this relationship may differ in autism from neurotypical peers.

Six other studies included different sensory domains in their analyses. Crasta et al. (2014) and Shamaya et al. (2017) reported multi‐modal associations between sensory processing and mealtime behaviours in autistic children, both similarly highlighting the significant role of oral sensory sensitivities. Furthermore, these two studies also found significant associations between emotional responses to sensory processing and mealtime behaviours, tentatively implicating the role of emotion in relationship between sensory processing and eating behaviours. Nadon et al. (2011) supported a multi‐sensory association with eating behaviours, however, an initially significant association with tactile sensitivities disappeared after adjusting for confounders of age, diagnostic category and comorbidities. This could mean that tactile sensitivities may be of less significance while other sensory sensitivities, such as taste/smell sensitivities, may be of greater significance in driving observed oral sensory sensitivities.

The remaining studies looking at mealtime behaviours highlighted the importance of taste/smell sensitivities. Padmanabhan and Schroff (2020) and Wang et al. (2014) reported multi‐modal correlations between sensory processing and mealtime behaviours, with the strongest associations observed for taste/smell sensitivities and stronger evidence reported in Wang et al. (2014)'s autism sample compared to neurotypical peers. Finally, Panerai et al. (2020) found that autistic children with feeding problems exhibited higher multi‐modal sensory differences compared to those without, with the largest effect size (0.52) being for taste/smell sensitivities. Furthermore, this was the only study that differentiated between hyper‐ and hypo‐sensitivity patterns and found significant differences between autistic children with feeding problems demonstrated greater impairments in hypersensitivities (Panerai et al., 2020). Both Padmanabhan and Schroff (2020) and Panerai et al. (2020) were deemed to be of high quality during the risk of bias assessment, suggesting that these studies provide strong evidence for the role of taste/smell sensitivities.

Two studies did not find significant evidence to support a relationship between sensory processing and mealtime behaviours. Zickgraf and Mayes (2018) implicated more physiological factors, such as appetite and constipation, while Bitsika and Sharpley (2018) found that restrictive and repetitive behaviours were the sole significant predictor of eating behaviours. However, both studies were assessed to have moderate to high risk of bias of measurement bias. Zickgraf and Mayes (2018) the study used a single measure for both sensory processing and eating behaviours, while Bitsika and Sharpley (2018) used an eating measure that had not been validated in their sample. Despite this, it should be noted the latter study conducted a robust analysis to remove overlap items between sensory and eating measures, allowing them to clearly investigate the link between the two.

### 4.2 Food selectivity or fussiness

Seven studies looked at food selectivity or fussiness as an eating outcome. Leader et al. (2020) reported that food selectivity, and not food refusal, was predicted by higher global sensory differences. Focussing on oral sensory sensitivities, Kral et al. (2015) found that autistic children with atypical oral sensitivities were significantly more likely to exhibit food selectivity than those with typical oral sensory processing. Zickgraf et al. (2020) and Schnizler (2014) similarly found oral sensitivities to emerge as independent, significant predictors of food selectivity, even when controlling for other modality sensitivities. Caution is warranted however, as these studies were deemed of poor quality due to inconsistent implementation of exposure and outcome measures (Zickgraf et al., 2020) and possible sampling bias (Schnizler, 2014).

Looking across different sensory domains, Pomoni (2016) reported multi‐modal associations with food selectivity, however this association was strongest with taste/smell sensitivities, being marginally stronger in the autism group (*r* = −0.834) compared to the neurotypical group (*r* = −8.24). This was supported by Smith et al. (2020), who included a neurotypical control group in their relationship analyses and reported that taste/smell sensitivities were not only an independent predictor of food fussiness but also an independent mediator of this relationship in autistic children and adolescents compared to their neurotypical peers. Conversely, one study did not report a significant relationship between sensory processing and food selectivity, reporting that children deemed to be selective eaters did not demonstrate significant differences in global sensory nor taste/smell processing (Tanner et al., 2015). This was found to be a high‐quality study, and therefore convincingly challenges the role of taste/smell sensitivities in food selectivity; however, it should be noted however that Tanner et al. (2015) did not include other sensory domains in their analyses and thus the significance of taste/smell sensitivities over other sensory modalities cannot be inferred.

### 4.3 Food refusal

Nine studies looked at the relationship between sensory processing and food refusal. Three studies looked at global sensory processing and food refusal, with two studies (Leader et al., 2021; Padmanabhan & Schroff, 2020) reporting significant associations. Leader et al. (2021) also included other eating behaviours in their regression and found that food refusal was the only eating outcome predicted by sensory processing scores. Conversely, Leader et al. (2021) did not find sensory scores to significantly predict food refusal, instead implicating food selectivity. Different samples and analyses were employed, with Leader et al. (2021) including a broader range of possible predictors and thus a possibly more robust regression analysis.

Looking at the relationship between food refusal and multi‐modal sensory processing, two studies reported significant associations with oral sensory processing (Chistol et al., 2018; Crasta et al., 2014). Chistol et al. (2018) also looked at hypersensitivities, reporting that the association between food refusal and oral sensory processing was strongest for autistic children with oral sensory hypersensitivities. Thus, oral sensory processing, and possibly oral hypersensitivities, may be significantly related to food refusal behaviours, although replication and future research is needed. Furthermore, Crasta et al. (2014) found significant associations between food refusal and other important sensory subscales, including the modulation of sensory input affecting emotion levels, a finding supported by Shamaya et al. (2017) who found significant associations between food refusal and modulation of visual input affecting emotion levels. Both studies highlight the importance of approaching food refusal as a multi‐dimensional sensory experience, related to differences across sensory, motor and emotional domains.

Three studies focussed on taste or smell sensitivities in relation to food refusal, reporting significant associations between food refusal and taste/smell sensitivities (Lane et al., 2014; Tanner et al., 2015). The latter study conducted further analysis using sensory subtypes developed in a previous study and reported that autistic children who exhibited the highest levels of food refusal displayed a complex sensory subtype characterised by taste/smell sensitivities, as well as proprioceptive dysfunction (see Lane et al., 2014). In the only study to use a physiological measure of sensory processing, Riccio et al. (2018) reported an association between the TARS238 gene proposed to be responsible for bitter taste perception and food refusal in autistic children at the limits of statistical significance (*p* = 0.07). Caution should be warranted here when interpreting these results as no power calculations were reported to justify their significance level, raising concerns of a reporting bias.

### 4.4 Ritualistic eating behaviours

Two studies looked at the relationship between sensory processing and ritualistic eating behaviours, reporting significant associations with behaviours such as ritualistic feeding patterns and rigid/perseverant eating (Martins et al., 2008; Pomoni, 2016). Pomoni (2016) also found that the strongest correlations were with taste/smell domains (*r* = −7.16). However, Martins et al. (2008) was assessed to reflect a high risk of measurement bias. Their measure of sensory processing was a single item response on the CARS, while their ritualistic feeding behaviour measure was developed for the study and was thus unvalidated. Therefore, due to the spare evidence base and possible bias concerns, findings are, at this stage, inconclusive.

### 4.5 Over‐ or under‐eating

Two studies looked at the relationship between sensory processing and over‐ or under‐eating. Koshy (2015) reported significant but weak (*r* = −0.228–0.122) correlations with both over‐ and under‐eating in autistic children and adolescents, while Kral et al. (2015) found that young autistic children with atypical oral sensitivities displayed greater emotional under‐eating, specifically due to negative emotions. This would imply that emotion may play a role in regulating the relationship between sensory processing and under‐eating. Under‐eating was implicated across both studies suggesting that this may be a common eating issue in autism, with tentative evidence to suggest this persist into adolescence.

## 5 FOOD REPERTOIRE

Seven studies included looked at food repertoire. Chistol et al. (2018) found oral sensitivities to be significantly associated with vegetable variety but not total repertoire or fruit variety. Interestingly, when the analysis focussed on oral hyper‐sensitivities, a significant relationship was observed across all three variables, thus highlighting the importance of distinguishing between hyper‐ and hypo‐sensitivities. Shamaya et al. (2017) reported a significant relationship between motor and visual domains, as well as taste/smell sensitivities and limited food repertoire. Tanner et al. (2015) and Lane et al. (2014) reported significant associations between taste/smell sensitivities and food repertoire, including a reduced number of total number of foods eaten and a limited variety of foods eaten. Tanner et al. (2015) only looked at taste/smell sensitivities, while Lane et al. (2014) included a broad range of sensory domains, thus suggesting that taste/smell sensitivities may play a particularly important role.

Conversely, one study (Suarez et al., 2012, 2014) reported that tactile hypersensitivity was significantly associated with limited food repertoire (Suarez et al., 2012), consistent across a follow up of 20 months (Suarez et al., 2014). Both papers were found to reflect a moderate risk of measurement bias, using eating behaviour measures that were developed for the purpose of the study and thus unvalidated. The study also adopted a dichotomous approach to sensory processing, looking at tactile hypersensitivities as one domain and putting all other senses in an 'other’ categories. Collectively, these studies suggest a limited food repertoire is associated with multi‐sensory differences, with more research required before any conclusions can be drawn regarding specific modalities.

## 6 FOOD NEOPHOBIA

Two studies investigated food neophobia, with both studies finding evidence to support a significant association. Kral et al. (2015) found that food neophobia was significantly elevated in autistic children with atypical oral sensitivities versus those with typical oral sensory processing, while Pomoni (2016) reported significant correlations between food neophobia and both taste/smell and tactile sensitivities, reporting strongest correlations with taste/smell (*r* = −0.589 vs. *r* = −0.312). Both studies also investigated this relationship in children between 4 and 14 years old, suggesting that this association may persist across childhood and into early adolescence. Collectively, these studies tentatively support a relationship between oral sensory processing and food neophobia, particularly taste/smell sensitivities, although they form a spare evidence base demanding future research, both with regards to the relationship itself and with regards to how this relationship persists across development.

## 7 DISCUSSION

The aim of this study was to provide a comprehensive synthesis of studies looking at the relationship between sensory processing and eating behaviours in autism. Included studies generally supported a significant relationship between sensory processing and a broad range of eating behaviours, with preliminary evidence to implicate taste/smell sensitivities and the possible role of hypersensitivities. Key findings are discussed below.

The relationship between sensory processing and eating behaviours was supported across all eating outcomes, suggesting that sensory processing underpins a broad range of eating behaviours in autism. An interesting finding from the current review was that only two studies investigated food neophobia (Kral et al., 2015; Pomoni, 2016), despite food neophobia being one of the most frequent eating behaviours in autism. This could be due to conceptual constraints of previous studies, reflecting heterogeneity of definitions and the conflation of food neophobia with food selectivity (Wallace et al., 2018). Future research should heed calls for independent measurement of food neophobia in order to generate a more focussed line of research and add to the limited evidence‐base regarding the relationship between food neophobia and sensory processing.

There was evidence across all three domains that eating was reflective of a multi‐sensory experience, with a broad range of sensory modalities implicated in the relationship with atypical eating behaviours. This is in line with previous research suggesting that over 90% of autistic individuals present with differences in sensory modulation, organisation and integration across multiple sensory domains (Ben‐Sasoon et al., 2009; Marco et al., 2011; Tomchek et al., 2007). These findings have clear implications regarding the design and implementation of mealtime interventions in autism, as they would suggest that detailed sensory evaluation and tailored sensory‐based interventions may be an effective way of managing atypical eating behaviours in autistic individuals. Such sensory‐based approaches to eating patterns and behaviours are starting to be incorporated into behavioural interventions, reporting promising results (Galpin et al., 2018; Luisier et al., 2015, 2019; Seiverling et al., 2018).

While oral sensory processing was associated with mealtime behaviours, food repertoire and food neophobia, it should be noted that oral processing encompasses multiple sensory domains, including taste/smell and tactile sensitivities (Chaware et al., 2021). The current review found evidence to suggest that tactile sensitivities may not play as significant a role, with studies either reporting non‐significant associations (Nadon et al., 2011; Zickgraf & Mayes, 2018) or having measurement limitations that prevent generalisable or comparable conclusions (e.g., Suarez et al., 2012, 2014). On the other hand, taste/smell sensitivities were consistently implicated in the relationship between general mealtime and eating behaviours (Padmanabhan & Schroff, 2020; Panerai et al., 2020; Wang et al., 2014), food selectivity (Pomoni, 2016; Smith et al., 2020), food refusal (Lane et al., 2014; Tanner et al., 2015), ritualistic eating behaviours (Pomoni, 2016), food repertoire (Lane et al., 2014; Tanner et al., 2015) and food neophobia (Pomoni, 2016), suggesting that taste/smell sensitivities may be an important underlying mechanism in the manifestation of a broad range of atypical eating behaviours.

A small number of studies implicated the role of hypersensitivities to sensory stimuli (Chistol et al., 2018; Suarez et al., 2012, 2014) however, at this stage, firm conclusions cannot be drawn. Only three studies investigated hypersensitivities in only two out of seven eating behaviours, reflecting a very small evidence base, and only one study (Panerai et al., 2020) included hypo‐sensitivities in their analysis. Intuitively, different sensory profiles may be associated with different eating difficulties; for example, hypersensitivities may be associated with food avoidance or restrictive behaviours while hyposensitivity's may lead to seeking sensations from food, which in turn may manifest as over‐eating. This latter hypothesis could help explain recent longitudinal evidence to suggest that fussy eating behaviours partially mediated the relationship between childhood autistic traits and disordered eating behaviours in adolescence, particularly binge eating behaviours (Leno et al., 2022). Further studies exploring different sensory profiles across a range of eating behaviours, including restrictive and binge eating, will allow researchers to address such hypotheses.

At the time of the literature search, no study looked at the relationship between sensory processing and disordered eating, despite research evidence to support an over‐representation of EDs, such as anorexia nervosa (AN; Westwood & Tchanturia, 2017) and Avoidant and Restrictive Food Intake Disorder (ARFID; Bourne et al., 2021). Elucidating underlying mechanisms of this relationship has clear clinical implications. For example, despite food‐specific sensory aversions being a diagnostic criterion of ARFID (APA, 2013), the majority of interventions have focussed on cognitive and behavioural techniques, and no study to date has looked at sensory‐based interventions in autism (Bourne et al., 2021). Similarly, there is a limited evidence base for treatment adaptions for autistic individuals with AN (Li et al., 2022), with only one clinical pathway identified (Tchanturia et al., 2020) that delivers promising but limited evidence of efficacy. A recent comprehensive framework of differences in common features of autism and AN include experiencing sensory processing difficulties as a prominent shared feature (Kinnaird & Tchanturia, 2021) and studies have begun to attempt to untangle these differences by exploring the role of sensory processing in AN samples with elevated autistic traits (e.g., Kinnaird et al., 2020), however his has yet to be explored in clinically diagnosed autistic samples. Encouragingly, a brief, pragmatic sensory screener implemented in the PEACE Pathway has been reported to be beneficial to eating disorder services in adjusting treatment to personal, sensory needs (Kinnaird et al., 2020), suggesting that sensory processing may play an important role and should be considered and adjusted for in clinical approaches to AN in autism.

Interestingly, the possible role of emotion was implicated across several eating behaviours, including mealtime behaviours and food refusal (Crasta et al., 2014; Shamaya et al., 2017). Specific emotions were investigated in one study only (Kral et al., 2015) which reported participants with oral sensitivities displayed greater emotional under‐eating, particularly due to negative emotions. These would tentatively suggest that experiencing emotion, particularly negative emotions, may lead some autistic individuals to reduce or restrict the amount of food they eat. While this is evidence base is limited and such hypotheses are exploratory at best, again the possible implications of this finding on the development of restrictive eating disorders, such as AN and ARFID, make it an interesting avenue for future research. Indeed, the underlying mechanisms of both comorbidities remain poorly understood. Similar sensory profiles have been reported in autism and in both ARFID and AN (Dovey et al., 2019; Kinnaird & Tchanturia, 2021), while differences in emotion processing (Brede et al., 2020; Courty et al., 2013; Kerr‐Gaffney et al., 2021) or alexithymia (Vuiller et al., 2020) have been proposed as possible mechanisms linking autism and AN. However, no study to date has explored the possible interplay between sensory processing, emotion and eating behaviours in the development of disordered eating in autism. Understanding the complex relationship between these domains may help researchers and clinicians work towards untangling mechanisms of these co‐existing conditions.

There were several methodological limitations observed across studies. There was a notable lack of control or comparison groups, with many studies excluding these groups in their relationship analyses, and almost all studies adopting a cross‐sectional design. Furthermore, the majority of studies conducting their research in Western countries and parent demographics typically skewed towards educated mothers, raising generalisability concerns. Almost all studies utilised parent‐report measures, reflecting an urgent need for direct, observational assessment of eating measures and physiological measures of sensory processing to eliminate bias and increase objectivity. Finally, while taste/smell sensitivities appear to be implicated, these two modalities are collapsed into one domain on the Sensory Profile and the Short Sensory Profile. It could be that one modality may play a more prominent role than the other, however, their conflation into one subscale leads to an inability to differentiate between their possible roles. Overall, only 6 out of 25 included studies were deemed to be of good quality, suggesting that these limitations may be raising notable bias concerns.

There were also several conceptual limitations. There was a notable lack of attention paid to specific sensory patterns, with only one study looking at hyposensitivity's (Panerai et al., 2020) and no study looking at multi‐sensory integration. Furthermore, no single study looked at adult participants, reflective of a wider childhood‐bias in autism research (Ratto & Mesibov, 2015), which may also in part explain why no study explored disordered eating as an outcome. Applying a developmental approach will shed important insights on the relationship between sensory processing and eating behaviours across the lifespan and may allow for the identification of early behaviours indicative of future disordered eating. See Table 4 for a summary of directions for future research highlighted in the current review.

**TABLE 4:** Summary of directions for future research

Conceptual considerations	Research design	Measurement and assessment	Interventions
Research focussing on food neophobia, including treatment of food neophobia as a distinct eating outcome	Research including control and/or comparison groups	Research adopting direct, observational assessments of eating behaviours	Research focussing on developing sensory‐based eating and feeding interventions for non‐clinical eating outcomes
Research focussing on disordered eating outcomes (restrict, binge, purge) in clinically diagnosed autism samples	Research adopting a longitudinal design, measuring the relationship across and/or development	Research adopting physiological or neurological assessments of sensory processing	Research focussing on developing sensory‐based eating and feeding interventions for disordered eating outcomes
Research focussing on taste and smell sensitivities as distinct sensory modalities	Research including more diverse and representative samples		
Research focussing on different sensory patterns, particularly hypersensitivities, hyposensitivities and sensory seeking	Research including more autistic adolescents and, in particular, autistic adults		
Research focussing on the possible role of emotion in the relationship between sensory processing and eating behaviours, particularly with regards to the development of disordered eating			

## 8 CONCLUSIONS

Understanding the relationship between sensory processing and eating behaviours in autism has important implications for clinicians and healthcare professionals, suggesting that individual sensory evaluation and tailored sensory interventions may help tackle a broad range of atypical eating behaviours. Careful consideration should be given to the possible importance of taste/smell sensitivities and hypersensitivities, however all possible sensory modalities and patterns should be evaluated and addressed. Elucidating the precise nature of this relationship is of the utmost importance in helping autistic individuals manage their eating behaviours and could even prevent the development of disordered eating. Future research should address the outlined methodological and conceptual limitations, working towards clear, standardized definitions of eating behaviours, and utilising longitudinal designs with more objective, physiological measures of sensory processing and eating behaviours.

## CONFLICT OF INTEREST

There are no conflict of interests associated with this review.

## Data Availability

Data sharing is not applicable to this article as no new data were created or analyzed in this study.

## References

[erv2920-bib-0001] Ahearn, W. H. , Castine, T. , Nault, K. , & Green, G. (2001). An assessment of food acceptance in children with autism or pervasive developmental disorder‐not‐otherwise‐specified. Journal of Autism and Developmental Disorders, 31(5), 505–511. 11794415 10.1023/a:1012221026124

[erv2920-bib-0002] American Psychiatric Association . (2013). Diagnostic and statistical manual of mental disorders (5th ed.). American Psychiatric Publishing.

[erv2920-bib-0003] Bandini, L. G. , Anderson, S. E. , Curtin, C. , Cermak, S. , Evans, E. W. , Scampini, R. , & Must, A. (2010). Food selectivity in children with autism spectrum disorders and typically developing children. The Journal of Pediatrics, 157, 259–264. 20362301 10.1016/j.jpeds.2010.02.013 PMC2936505

[erv2920-bib-0004] Bandini, L. G. , Curtin, C. , Philips, S. , Anderson, S. E. , Maslin, M. , & Must, A. (2017). Changes in food selectivity in children with autism spectrum disorder. Journal of Autism and Developmental Disorders, 47(2), 439–446. 27866350 10.1007/s10803-016-2963-6 PMC5310968

[erv2920-bib-0005] Baranek, G. T. , David, F. J. , Poe, M. D. , Stone, W. L. , & Watson, L. R. (2006). Sensory experiences Questionnaire: Discriminating sensory features in young children with autism, developmental delays, and typical development. Journal of Child Psychology and Psychiatry, 47(6), 591–601. 16712636 10.1111/j.1469-7610.2005.01546.x

[erv2920-bib-0006] Baraskewich, J. , von Ranson, K. M. , McCrimmon, A. , & McMorris, C. A. (2021). Feeding and eating problems in children and adolescents with autism: A scoping review. Autism, 25(6), 1505–1519. 33653157 10.1177/1362361321995631 PMC8323334

[erv2920-bib-0007] Ben‐Sasoon, A. , Hen, L. , Fluss, R. , Cermak, S. A. , Engel‐Yeger, B. , & Gal, E. (2009). A meta‐analysis of sensory modulation symptoms in individuals with autism spectrum disorders. Journal of Autism and Developmental Disorders, 39, 1–11. 18512135 10.1007/s10803-008-0593-3

[erv2920-bib-0008] Bitsika, V. , & Sharpley, C. F. (2018). Specific aspects of repetitive and restricted behaviours are of greater significant than sensory processing difficulties in eating disorders in high functioning young girls with ASD. Journal of Developmental Disability, 30, 259–267.

[erv2920-bib-0009] Bourne, L. , Mandy, W. , & Bryant‐Waugh, R. (2021). Avoidant/restrictive food intake disorder and severe food selectivity in children and young people with autism. Developmental Medicine & Child Neurology. Retrieved from 10.1111/dmcn.15139 35112345

[erv2920-bib-0010] Boyd, B. A. , McBee, M. , Holtzclaw, T. , Baranek, G. T. , & Bodfish, J. W. (2009). Relationships among repetitive behaivours, sensory features and executive functions in high functioning autism. Research in Autism Spectrum Disorders, 3(4), 959–966. 21475640 10.1016/j.rasd.2009.05.003 PMC3071047

[erv2920-bib-0011] Brede, J. , Babb, C. , Jones, C. , Elliott, M. , Zanker, C. , Mandy, W. , Serpell, L. , Fox, J. , & Mandy, W. (2020). For me, the anorexia is just a symptom, and the cause is autism”: Investigating restrictive eating disorders in autistic women. Journal of Autism and Developmental Disorders, 50, 4280–4296. 32274604 10.1007/s10803-020-04479-3 PMC7677288

[erv2920-bib-0012] Brown, C. L. , Vander Schaaf, E. B. , Cohen, G. M. , Irby, M. B. , & Skelton, J. A. (2016). Association of picky eating and food neophobia with weight: A systematic review. Childhood Obesity, 12(4), 247–262. 27135525 10.1089/chi.2015.0189 PMC4964761

[erv2920-bib-0013] Cermak, S. A. , Curtin, C. , & Bandini, L. G. (2010). Food selectivity and sensory sensitivity in children with autism spectrum dis‐ orders. Journal of the American Dietetic Association, 110(2), 238–246. 20102851 10.1016/j.jada.2009.10.032 PMC3601920

[erv2920-bib-0014] Chaware, S. H. , Dubey, S. G. , Katatkar, V. , Jankar, A. , Pustake, S. , & Darekar, A. (2021). The systematic review and meta‐analysis of oral sensory challenges in children and adolescents with autism spectrum disorders. Journal of International Society of Preventive and Community Dentistry, 11(5), 469–480. 34760790 10.4103/jispcd.JISPCD_135_21 PMC8533039

[erv2920-bib-0015] Chistol, L. , Bandini, L. , Must, A. , Phillips, S. , Cermak, S. , & Curtin, C. (2018). Sensory sensitvity and food selectivity in children with autism spectrum disorder. Journal of Autism and Developmental Disorders, 48(2), 583–591. 29116421 10.1007/s10803-017-3340-9 PMC6215327

[erv2920-bib-0083] Courty, A. , Maria, A. S. , Lalanne, C. , Ringuenet, D. , Vindreau, C. , Chevallier, C. , Pouga, L. , Pinabel, F. , Philippe, A. , Adrien, J.‐L. , Barry, C. , & Berthoz, S. (2013). Levels of autistic traits in anorexia nervosa: A comparative psychometrics study. BMC Psychiatry, 13(222). 10.1186/1471-244X-13-222 PMC3848448 24015680

[erv2920-bib-0016] Crasta, J. E. , Benjamin, T. E. , Suresh, A. P. C. , Alwinesh, M. T. J. , Kanniappan, G. , & Nair, M. K. C. (2014). Feeding problems among children with autism in a clinical population in India. Indian Journal of Pediatrics, 81(2), 169–172. 10.1007/s12098-014-1630-1 25413215

[erv2920-bib-0017] Crasta, J. E. , Salzinger, J. E. , Lin, M. , Gavin, W. J. , & Davies, P. L. (2020). Sensory processing and attention profiles among children with sensory processing disorders and autism spectrum disorders. Frontiers in Integrative Neuroscience, 22:14. 10.3389/fnint.2020.00022 PMC7214749 32431600

[erv2920-bib-0018] De Both, K. K. , & Reynolds, S. (2015). A systematic review of sensory‐based autism subtypes. Research in Autim Spectrum Disorders, 4(36), 44–56.

[erv2920-bib-0019] DeMand, A. , Johnson, C. , & Foldes, E. (2015). Psychometric properties of the brief autism mealtime behaviours inventory. Journal of Autism and Developmental Disorders, 45(9), 2667–2673. 25813517 10.1007/s10803-015-2435-4 PMC4554795

[erv2920-bib-0079] Dovey, L. M. , Kumari, V. , Blissett, J. , & Mealtime Parent Science Gang . (2019). Eating behaviour, behavioural problems and sensory profiles of children with avoidant/restrictive food intake disorder (ARFID), autism spectrum disorders or picky eating: Same or different? European Psychiatry, 61, 56–62. 31310945 10.1016/j.eurpsy.2019.06.008

[erv2920-bib-0020] Dovey, T. M. , Staples, P. A. , Gibson, E. L. , & Halford, J. C. (2008). Food neophobia and ‘picky/fussy’ eating in children: A review. Appetite, 50, 181–193. 17997196 10.1016/j.appet.2007.09.009

[erv2920-bib-0021] Dubois, L. , Farmer, A. P. , Girard, M. , & Peterson, K. (2008). Preschool children's eating behaviors are related to dietary adequacy and body weight. European Journal of Clinical Nutrition, 61, 846–855. 10.1038/sj.ejcn.1602586 17180152

[erv2920-bib-0022] Edmond, A. , Emmet, P. , Steer, C. , & Golding, J. (2010). Feeding symptoms, dietary patterns, and growth in young children with autism spectrum disorders. Pediatrics, 126(2), 337–342. 10.1542/peds.2009-2391 20643716

[erv2920-bib-0023] Galpin, J. , Osman, L. , & Paramore, C. (2018). Sensory snack time: A school‐based intervention addressing food selectivity in autistic children. Frontiers in Education, 3(77). 10.3389/feduc.2018.00077

[erv2920-bib-0024] Gesi, C. , Carmassi, C. , Luciano, M. , Bossini, L. , Ricca, V. , & Dell’Osso, L. (2021). Autistic traits in patients with anorexia nervosa, bulimia nervosa or binge eating disorder: A pilot study. European Psychiatry, 41(1). 10.1016/j.eurpsy.2017.01.310

[erv2920-bib-0025] Glod, M. , Riby, D. M. , Honey, E. , & Rodgers, J. (2015). Psychological correlates of sensory processing patterns in individuals with autism spectrum disorder: A systematic review. Review Journal of Autism and Developmental Disorders, 2, 199–221.

[erv2920-bib-0026] Hubbard, K. L. , Anderson, S. E. , Curtin, C. , Must, A. , & Bandini, L. G. (2014). A comparison of food refusal related to characteristics of food in children with autism spectrum disorders and typically developing children. Journal of the Academy of Nutrition and Dietetics, 114(12), 1981–1987. 24928779 10.1016/j.jand.2014.04.017 PMC4252256

[erv2920-bib-0080] Iarocci, G. , & McDonald, J. (2006). Sensory integration and the perceptual experience of persons with autism. Journal of Autism and Developmental Disorders, 36(1), 77–90. 16395537 10.1007/s10803-005-0044-3

[erv2920-bib-0081] Kerr‐Gaffney, J. , Hayward, H. , Jones, E. J. H. , Halls, D. , Murphy, D. , & Tchanturia, K. (2021). Autism symptoms in anorexia nervosa: A comparative study with females with autism spectrum disorder. Molecular Autism, 21(1). 10.1186/s13229-021-00455-5 PMC8247081 34193255

[erv2920-bib-0027] Kinnaird, E. , Dandil, D. , Li, Z. , Smith, K. , Pimblett, C. , Tchanturia, K. , Stewart, C. , & Tchanturia, K. (2020). Pragmatic sensory screening in anorexia nervosa and associations with autistic traits. Journal of Clinical Medicine, 9(4), 1182. 10.3390/jcm9041182 PMC7230430 32326069

[erv2920-bib-0028] Kinnaird, E. , Stewart, C. , & Tchanturia, K. (2018). Taste sensitivity in anorexia nervosa: A systematic review. International Journal of Eating Disorders, 51(8), 771–784. 29984498 10.1002/eat.22886 PMC6282513

[erv2920-bib-0029] Kinnaird, E. , Stewart, C. , & Tchanturia, K. (2020). The relationship of autistic traits to taste and olfactory processing in anorexia nervosa. Molecular Autism, 11(1), 25. 10.1186/s13229-020-00331-8 32276668 PMC7146886

[erv2920-bib-0030] Kinnaird, E. , & Tchanturia, K. (2021). Looking beneath the surface: Distinguishing between common features in autism and anorexia nervosa. Journal of Behavioural and Cognitive Therpay, 31(1), 3–13.

[erv2920-bib-0031] Koshy, B. (2015). The ASD‐plus study: Co‐existing emotional and behavioural conditions in children with autism spectrum disorder – frequency, severity and correlates from two large UK databases. Institute of Neuroscience, Newcastle University. Unpublished manuscript.

[erv2920-bib-0032] Kral, T. V. E. , Souders, M. C. , Tompkins, V. H. , Remiker, A. M. , Eriksen, W. T. , & Pinto‐Martin, J. A. (2015). Child eating behaviours and caregiver feeding practices in children with autism spectrum disorders. Public Health Nursing, 32(5), 488–497. 25112438 10.1111/phn.12146

[erv2920-bib-0033] Kuschner, E. S. , Eisenberg, I. W. , Orionzi, B. , Simmons, W. K. , Kenworthy, L. , Martin, A. , & Wallace, G. L. (2015). A preliminary study of self‐reported food selectivity in adolescents and young adults with autism spectrum disorder. Research in Autism Spectrum Disorders, 15, 53–59. 26309446 10.1016/j.rasd.2015.04.005 PMC4545503

[erv2920-bib-0034] Lane, A. E. , Geraghty, M. E. , Young, G. S. , & Rostorfer, J. L. (2014). Problem eating behaviours in autism spectrum disorder are associated with suboptimal daily nutrient intake and taste/smell sensitivity. Clinical Research Reports, 6(3), 172–180.

[erv2920-bib-0035] Leader, G. , O’Reilly, M. , Gilroy, S. P. , Chen, J. L. , Ferrari, C. , & Mannion, A. (2021). Comorbid feeding and gastrointestinal symptoms, challenging behaviour, sensory issues, adaptive functioning and quality of life in children and adolescents with autism spectrum disorder. Developmental Neurorehabilitation, 24(1), 35–44. 32496834 10.1080/17518423.2020.1770354

[erv2920-bib-0036] Leader, G. , Tuohy, E. , Chen, J. L. , Mannion, A. , & Gilroy, S. P. (2020). Feeding problems, gastrointenstinal symptoms, challenging behaviour and sensory issues in children and adolescents with autism spectrum disorder. Journal of Autism and Developmental Disorders, 50(4), 1401–1410. 31955310 10.1007/s10803-019-04357-7

[erv2920-bib-0037] Leno, V. C. , Micali, N. , Bryant‐Waugh, R. , & Herle, M. (2022). Associations between childhood autistic traits and adolescent eating disorder behaviours are partially mediated by fussy eating. European Eating Disorders Review. 10.1002/erv.2902 PMC9542277 35388530

[erv2920-bib-0038] Li, Z. , Halls, D. , Tchanturia, K. , & Byford, S. (2022). Autistic characteristics in eating disorders: Treatment adaptions and impact on clinical outcomes. European Eating Disorders Review. 10.1002/erv.2875 34850503

[erv2920-bib-0039] Luisier, A. , Petitpierre, G. , Berod, A. C. , Richoz, A. , Lao, J. , Caldara, R. , & Bensafi, M. (2019). Visual and hedonic perception of food stimuli in children with autism spectrum disorders and their relationship to food neophobia. Perception, 48(3), 197–213. 30758252 10.1177/0301006619828300

[erv2920-bib-0040] Luisier, A. , Petitpierre, G. , Ferdenzi, C. , Berod, A. C. , Giboreau, A. , Rouby, C. , & Bensafi, M. (2015). Odor perception in children with autism spectrum disorder and its relationship to food neophobia. Frontiers in Psychology, 6, 1830. 10.3389/fpsyg.2015.01830 26648891 PMC4664613

[erv2920-bib-0041] Marco, E. J. , Hinkley, L. B. , Hill, S. S. , & Nagarajan, S. S. (2011). Sensory processing in autism: A review of neurophysiologic findings. Pediatric Research, 69(5 Pt2), 48–54. 10.1203/PDR.0b013e3182130c54 PMC3086654 21289533

[erv2920-bib-0042] Margari, L. , Marzulli, L. , Gabellone, A. , & de Giambattista, C. (2020). Eating and mealtime behaviours in patients with autism spectrum disorder: Current perspectives. Neuropsychiatric Disease and Treatment, 16, 2083–2102. 32982247 10.2147/NDT.S224779 PMC7504729

[erv2920-bib-0043] Mari‐Bauset, S. , Zazpe, I. , Mari‐Sanchis, A. , Llopis‐Gonzalez, A. , & Morales‐Suarez‐Varela, M. (2014). Food selectivity in autism spectrum disorders: A systematic review. Journal of Child Neurology, 29(11), 1554–1561. 24097852 10.1177/0883073813498821

[erv2920-bib-0045] Martins, Y. , Young, R. L. , & Robson, D. C. (2008). Feeding and eating behaviours in children with autism and typically developing peers. Journal of Autism and Developmental Disorders, 38, 1878–1887. 18483843 10.1007/s10803-008-0583-5

[erv2920-bib-0046] Micali, N. , Simonoff, E. , Elberling, H. , Rask, C. U. , Olsen, E. M. , & Skovgaard, A. M. (2011). Eating patterns in a population‐ based sample of children aged 5 to 7 years: Association with psychopathology and parentally perceived impairment. Journal of Developmental and Behavioural Paediatrics, 32, 572–580. 10.1097/DBP.0b013e31822bc7b7 21918471

[erv2920-bib-0047] Nadon, G. , Feldman, D. E. , Dunn, W. , & Gisel, E. (2011). Association of sensory processing and eating problems in children with autism spectrum disorders. Autism Research and Treatment, 2011(541926), 2011–2018. 10.1155/2011/541926 PMC3420765 22937249

[erv2920-bib-0048] Padmanabhan, P. S. , & Schroff, H. (2020). The relationship between sensory integration challenges and the dietary intake and nutritional status of children with autism spectrum disorders in Mumbai, India. International Journal of Developmental Disabilities, 66(2), 142–152. 10.1080/20473869.2018.1522816 PMC8132926 34141376

[erv2920-bib-0049] Page, M. , McKenzie, J. E. , Bossuyt, M. C. , Boutron, I. , Hoffman, T. C. , & Moher, D. (2020). The PRISMA 2020 statement: An updated guideline for reporting systematic review. BMJ, 372. 10.1136/bmj.n71 PMC8005924 33782057

[erv2920-bib-0050] Page, S. D. , Souders, M. C. , Kral, T. V. E. , Chao, A. M. , & Pinto‐Martin, J. (2021). Correlates of feeding difficulties among children with autism spectrum disorder: A systematic review. Journal of Autism and Developmental Disorders, 52(1), 255–274. 33666799 10.1007/s10803-021-04947-4

[erv2920-bib-0051] Panerai, S. , Ferri, R. , Catania, V. , Zingale, M. , Ruccella, D. , Elia, M. , Fasciana, D. , & Elia, M. (2020). Sensory profiles of children with autism spectrum disorder with and without feedinb problems: A comparative study in Sicilian subjects. Brain Sciences, 10(6), 336. 10.3390/brainsci10060336 PMC7349225 32486513

[erv2920-bib-0052] Pomoni, M. (2016). Factors affecting the eating behaviour of individuals with and without autism spectrum conditions. School of Psychology, College of Life and Environmental Sciences, University of Birmingham. Unpublished manuscript.

[erv2920-bib-0053] Postorino, V. , Sanges, V. , Giovagnoli, G. , Fatta, L. M. , De Peppo, L. , Mazzone, L. , Vicari, S. , & Mazzone, L. (2015). Clinical differences in children with autism spectrum disorder with and without food selectivity. Appetitive, 92, 126–132. 10.1016/j.appet.2015.05.016 25998237

[erv2920-bib-0054] Ratto, A. B. , & Mesibov, G. B. (2015). Autism spectrum disorders in adolescence and adulthood: Long‐term outcomes and relevant issues for treatment and research. Science China Life Sciences, 58(10), 1010–1015. 26335732 10.1007/s11427-012-4295-x

[erv2920-bib-0055] Riccio, M. P. , Franco, C. , Negri, R. , Ferrentino, R. I. , Maresca, R. , Bravaccio, C. , Greco, L. , & Bravaccio, C. (2018). Is food refusal in autistic children related to TAS2R38 genotype? Autism Research, 11(3), 531–538. 29282878 10.1002/aur.1912

[erv2920-bib-0056] Robertson, C. E. , & Baron‐Cohen, S. (2017). Sensory perception in autism. Nature Reviews Neuroscience, 18(11), 671–684. 28951611 10.1038/nrn.2017.112

[erv2920-bib-0057] Schnizler, A. (2014). Aberrant eating behaviours in children with autism spectrum disorder and their correlates. College of Arts and Sciences, University of Virginia. Unpublished manuscript.

[erv2920-bib-0058] Schrek, K. A. , & Williams, K. (2006). Food preferences and factors influencing food selectivity for children with autism spectrum disorders. Research in Developmental Disabilities, 27, 353–363. 16043324 10.1016/j.ridd.2005.03.005

[erv2920-bib-0059] Schrek, K. A. , Williams, K. , & Smith, A. F. (2004). A comparison of eating behaviours between children with and without autism. Journal of Autism and Developmental Disorders, 34, 433–438. 15449518 10.1023/b:jadd.0000037419.78531.86

[erv2920-bib-0060] Seiverling, L. , Anderson, K. , Rogan, C. , Almaio, C. , Argott, P. J. , & Panora, J. (2018). A comparison of a behavioural feeding intervention with and without pre‐meal sensory integration therapy. Journal of Autism and Developmental Disorders, 48(10), 3344–3353. 29744703 10.1007/s10803-018-3604-z

[erv2920-bib-0061] Shamaya, Y. , Eilar‐Adar, S. , Leitner, Y. , Reif, S. , & Gabis, L. V. (2017). Meal time behaviour difficulties but not nutritional deficiencies correlate with sensory processing in children with autism spectrum disorder. Research in Developmental Disabilities, 66, 27–33. 28578072 10.1016/j.ridd.2017.05.004

[erv2920-bib-0062] Sharp, W. G. , Berry, R. C. , McCracken, C. , Nuhu, N. N. , Marvel, E. , & Jaquess, D. L. (2013). Feeding problems and nutrient intake in children with autism spectrum disorders: A meta‐analysis and comprehensive review of the literature. Journal of Autism and Developmental Disorders, 43(3), 2159–2173. 23371510 10.1007/s10803-013-1771-5

[erv2920-bib-0063] Smith, B. , Rogers, S. L. , Blissett, J. , & Ludlow, A. K. (2020). The relationship between sensory sensitivity, food fussiness and food preferences in children with neurodevelopmental disorders. Appetite, 150. 10.1016/j.appet.2020.104643 32105808

[erv2920-bib-0064] Solmi, F. , Bentivegna, F. , Bould, H. , Mandy, W. , Kothari, R. , Lewis, G. , Skuse, D. , & Lewis, G. (2021). Trajectories of autistic social traits in childhood and adolescence and disordered eating behaviours at age 14 years: A UK general population cohort study. Journal of Child Psychology and Psychiatry, 62(1), 75–85. 32361997 10.1111/jcpp.13255 PMC8425328

[erv2920-bib-0084] Stevenson, R. A. , Siemann, J. K. , Schneider, B. C. , Eberly, H. E. , Woynarkoski, T. G. , Camarata, S. M. , & Wallace, M. T. (2015). Multisensory integration in autism spectrum disorders. The Journal of Neuroscience, 34(3), 691–697. 10.1523/JNEUROSCI.3615-13.2014 PMC3891950 24431427

[erv2920-bib-0065] Suarez, M. A. , Nelson, N. W. , & Curtis, A. B. (2012). Associations of physiological factors, age and sensory over‐responsivity with food selectivity in children with autism spectrum disorders. The Open Journal of Occupational Therapy, 1(2). 10.15453/2168-6408-1004

[erv2920-bib-0066] Suarez, M. A. , Nelson, N. W. , & Curtis, A. B. (2014). Longitudinal follow‐up of factors associated with food selectivity in children with autism spectrum disorders. Autism, 18(8), 924–932. 24121181 10.1177/1362361313499457

[erv2920-bib-0067] Tanner, K. , Case‐Smith, J. , Nihikian‐Nelms, M. , Ratliff‐Schaub, K. , Spees, C. , & Carragh, A. R. (2015). Behavioural and physiological factors associated with selective eating in children with autism spectrum disorder. American Journal of Occupational Therapy, 69(9). 10.5014/ajot.2015.019273 PMC4643377 26565096

[erv2920-bib-0068] Tchanturia, K. , Adamson, J. , Leppanen, J. , & Westwood, H. (2019). Characteristics of autism spectrum disorder in anorexia nervosa: A naturalistic study in an inpatient treatment programme. Autism, 23(1), 123–130. 29105513 10.1177/1362361317722431

[erv2920-bib-0069] Tchanturia, K. , Smith, K. , Glennon, D. , & Burhouse, A. (2020). Towards an improved understanding of the anorexia nervosa and autism spectrum comorbidity: PEACE pathway implementation. Frontiers in Psychiatry, 11. 10.3389/fpsyt.2020.006400 PMC7358367 32733294

[erv2920-bib-0078] Tomchek S, D. , & Dunn W. (2007). Sensory Processing in Children With and Without Autism: A Comparative Study Using the Short Sensory Profile. The American Journal of Occupational Therapy, 61(2), 190–200. 17436841 10.5014/ajot.61.2.190

[erv2920-bib-0070] Trinh, E. (2014). Parent and child factors in relation to mealtime problem behaviour of children with and without autism spectrum disorder. The University of Alabama. Unpublished manuscript.

[erv2920-bib-0071] Twachtman‐Reilly, J. , Amaral, S. C. , & Zebrownki, P. P. (2008). Addressing feeding disorders in children on the autism spectrum in school‐based settings: Physiological behavioural issues. Language, Speech, and Hearing Services in Schools, 39(2), 261–272. 18420528 10.1044/0161-1461(2008/025)

[erv2920-bib-0082] Vuillier, L. , Carter, Z. , Teixeira, A. R. , & Moseley, R. L. (2020). Alexithymia may explain the relationship between autistic traits and eating disorder pathology. Molecular Autism, 63(11). 10.1186/s13229-020-00364-z PMC7406391 32758290

[erv2920-bib-0072] Wallace, G. L. , Llewellyn, C. , Fildes, A. , & Ronald, A. (2018). Autism spectrum disorder and food neophobia: Clinical and subclinical links. Journal of Nutrition, 108(4), 701–707. 10.1093/ajcn/nqy163 30321276

[erv2920-bib-0073] Wang, G. , Li, W. , Han, Y. , Dai, W. , Su, Y. , Zhang, X. , & Zhang, X. (2019). Sensory processing problems and comorbidities in Chinese preschool children with autism spectrum disorders. Journal of Autism and Developmental Disorders, 49(10), 4097–4108. 31267289 10.1007/s10803-019-04125-7

[erv2920-bib-0074] Westwood, H. , & Tchanturia, K. (2017). Autism spectrum disorder in anorexia nervosa: An updated literature review. Current Psychiatric Reports, 19(7), 41. 10.1007/s11920-017-0791-9 PMC5443871 28540593

[erv2920-bib-0075] Zickgraf, H. , & Mayes, S. D. (2018). Psychological, Health and Demographic correlates of atypical eating behaviours in children with autism. Journal of Developmental and Physical Disabilities, 31(3), 399–418.

[erv2920-bib-0076] Zickgraf, H. , Richard, E. , Zucker, N. L. , & Wallace, G. L. (2020). Rigidity and sensory sensitivity: Independent contributions to selective eating in children, adolescents and young adults. Journal of Clinical Child and Adolescent Psychology, 19, 1–13. 10.1080/15374416.2020.1738236 32189525

[erv2920-bib-0077] Zobel‐Lachuisa, J. , Andrianopoulos, M. V. , Mallioux, Z. , & Cermak, S. A. (2015). Sensory differences and mealtime behaviour in children with autism. American Journal of Occupational Therapy, 69(5), 6905185050p1–6905185050p8. 10.5014/ajot.2015.016790 PMC4572838 26379266

